# Complete Genome Sequences of Three *Limnohabitans* sp. (Lhab-A3) Strains, INBF002, TEGF004, and MORI2, Isolated from Two Lakes and a River in Japan

**DOI:** 10.1128/mra.01296-22

**Published:** 2023-02-22

**Authors:** Yusuke Ogata, Keiji Watanabe, Shusuke Takemine, Chie Shindo, Rina Kurokawa, Wataru Suda

**Affiliations:** a RIKEN Center for Integrative Medical Sciences, Yokohama, Kanagawa, Japan; b Center for Environmental Science in Saitama, Kazo, Saitama, Japan; University of Southern California

## Abstract

Freshwater bacterioplankton of the genus *Limnohabitans* represent a dominant group that has worldwide distribution. Here, we report the complete genome sequences of three *Limnohabitans* sp. (Lhab-A3 tribe) strains, i.e., INBF002, TEGF004, and MORI2, which were isolated from surface water samples from two shallow eutrophic lakes and a river in Japan.

## ANNOUNCEMENT

Freshwater bacterioplankton of the genus *Limnohabitans* of the Lhab-A3 tribe (LimA lineage) are known to be a cosmopolitan group with sometimes relatively high abundance (1–3). To date, the Lhab-A3 tribe contains only two species (Limnohabitans australis and Limnohabitans curvus) (2). Accumulation of genomic information on Lhab-A3 will contribute to insights into the physiochemical and ecological processes of the tribe.

Here, we report the complete genome sequences of three *Limnohabitans* sp. (Lhab-A3) strains, namely, INBF002 (JCM 16620), TEGF004 (JCM 16621), and MORI2 (JCM 32223), which were isolated from samples that had been collected from the surface water (depth of 0 to 50 cm) of two shallow eutrophic lakes, i.e., Lake Inbanuma, Inzai, Chiba, Japan (35°47′33.0″N, 140°14′58.8″E), and Lake Teganuma, Kashiwa, Chiba, Japan (35°51′31.8″N, 140°01′ 25.8″E), on 23 January 2008 and Karasawa River, Fukaya, Saitama, Japan (36°12′49.9″N, 139°17′27.0″E), on 4 September 2014. The 10-mL samples were filtered through a disposable syringe equipped with a 0.7-μm particle-retention glass-fiber filter (Whatman Puradisc 25 GF/F). After filtration, 100-μL aliquots of the filtrates were spread on modified Reasoner’s 2A (MR2A) agar plates and incubated at 25°C for 2 days (4). A single bacterial colony for each strain was picked, inoculated into sterilized MR2A liquid medium (pH 7.2), and incubated at 25°C for 1 day with reciprocal shaking (120 rpm), and pure strain cell suspensions were preserved at −80°C as stocks in MR2A broth supplemented with 20% (wt/vol) glycerol. Cells from the glycerol stocks were inoculated and cultured in MR2A liquid medium, harvested by centrifugation, and used for genomic DNA extraction.

**FIG 1:**
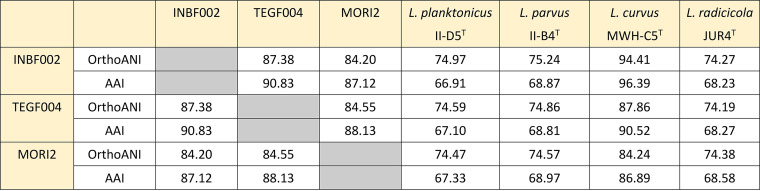
OrthoANI and AAI values for INBF002, TEGF004, MORI2, and four *Limnohabitans* type strains.

Genomic DNA was extracted from strains INBF002, TEGF004, and MORI2 using enzymatic lysis and phenol-chloroform-isoamyl alcohol extraction as described previously (5). Whole-genome sequencing was performed using a Sequel II system (Pacific Biosciences [PacBio]). The library was prepared with the SMRTbell Express template preparation kit v2.0 (PacBio), with DNA shearing with g-TUBEs (Covaris) and with target lengths of 10 to 15 kb. The PacBio reads were converted to circular consensus sequencing (CCS) reads using CCS software v6.2.0 (https://github.com/PacificBiosciences/ccs) and assembled using the assembler Canu v2.1.1 with specified parameters (minReadLength=2200, minOverlapLength=2200) (6), and the generated contig was checked for circularization to remap the CCS reads by minimap2 v2.24-r1122 (7). The genomic sequence obtained was annotated and rotated to start at *dnaA* using DFAST (https://dfast.nig.ac.jp) (8). Default parameters were used for all software analysis unless otherwise specified. The reads obtained and the genomic sequences are summarized in Table 1. The small circular contig of strain TEGF004 had two plasmid stabilization proteins (LTEGF4_25570 and LTEGF4_26240), with a plasmid probability score of 0.29 determined by PlasClass software (updated 30 October 2021) (9). The average nucleotide identity by orthology (OrthoANI) values determined using the Orthologous ANI Tool (OAT) (updated 14 June 2017) (10) and the average amino acid identity (AAI) values (http://enve-omics.ce.gatech.edu/aai) (11) of INBF002, TEGF004, MORI2, and four *Limnohabitans* type strains are shown in Fig. 1. The three isolates were closest to L. curvus MWH-C5^T^. The tribe assignment of the isolates was performed with the freshwater TaxAss 16S rRNA databases (FreshTrain 15 June 2020 release and SILVA v138) and pipeline (https://github.com/McMahonLab/TaxAss) using the 16S rRNA gene sequences obtained from genome sequences (12). The three isolates were assigned to the Lhab-A3 tribe. Thus, strains INBF002, TEGF004, and MORI2 belong to *Limnohabitans* sp. of the Lhab-A3 tribe.

**TABLE 1:** Information on the reads and contigs obtained for *Limnohabitans* sp. (Lhab-A3) strains INBF002, TEGF004, and MORI2

Parameter	Data for strain:		
	INBF002	TEGF004	MORI2
Data for quality-checked Sequel reads			
No. of reads	30,000	14,514	3,618
Total no. of bases	402,414,817	220,303,489	53,027,782
*N*_50_ (bp)	13,228	15,154	14,608
Total no. of contigs			
Chromosome	1	1	1
Plasmid	0	1	0
BioProject accession no.	PRJDB14571	PRJDB14571	PRJDB14571
BioSample accession no.	SAMD00549750	SAMD00549751	SAMD00549752
DRA accession no.	DRR413434	DRR413435	DRR413436
Chromosome features			
Genome size (bp)	2,594,181	2,624,462	2,649,037
GC content (%)	56.4	55.9	55.3
GenBank/ENA/DDBJ accession no.	AP027055	AP027056	AP027058
Plasmid features			
Genome size (bp)		148,919	
GC content (%)		49.7	
GenBank/ENA/DDBJ accession no.		AP027057	
Genome coverage (×)	155	79	20

### Data availability.

The genome sequences and raw data are available in GenBank/ENA/DDBJ under the BioProject accession number PRJDB14571. The BioSample, DDBJ Sequence Read Archive (DRA), and DDBJ accession numbers are provided in Table 1.
